# Investigating the Design and Implementation of an In-Line Near-Infrared Probe Using Computational Fluid Dynamics for Measurement of Non-Newtonian Fluids

**DOI:** 10.1177/00037028211062239

**Published:** 2022-02-10

**Authors:** Kiran Haroon, Thomas John, Cláudio P. Fonte, Ćesar Mendoza, Michael Baker, Philip Martin

**Affiliations:** 1 Department of Chemical Engineering & Analytical Science, 5292 University of Manchester, Manchester, UK; 2 63784 Unilever R&D, Port Sunlight, Bebington, UK

**Keywords:** Near-infrared, NIR, computational fluid dynamics, CFD, process probe, optimization

## Abstract

Process analytical technology (PAT) has developed significantly since its introduction in pharma where many in situ analytical probes and measuring devices are now commercially available, replacing the use of off-line quality control measurements that are typically laborious and time intensive. The use of PAT instrumentation should not interfere with the process itself and subsequently should have no effect on the product whilst measuring representative samples. Implementation of these devices is typically arbitrary using empirical means. Therefore, the objective of this study is to highlight the use of computational fluid dynamics modeling to investigate the effect of interfacing parameters and process parameters of an inline near-infrared (NIR) probe used to determine the viscosity of a non-Newtonian micellar liquid. The parameters investigated for the probe were immersion depth, immersion angle, gap size, and fluid velocity. The results conclude that the immersion angle and depth should both be optimized to prevent stagnant fluid accumulating in the measuring gap ensuring that the NIR measurements are representative of the bulk. The gap size determines the optical pathlength and therefore was also investigated against an existing predictive viscosity model showing no changes in model performance with varying gap size. The use of computational modeling to develop a digital twin prior to PAT implementation at the equipment design stage ensures the technology can perform at its best and will also aid in calibration transfer studies.

## Graphical abstract

**Graphical Abstract:**
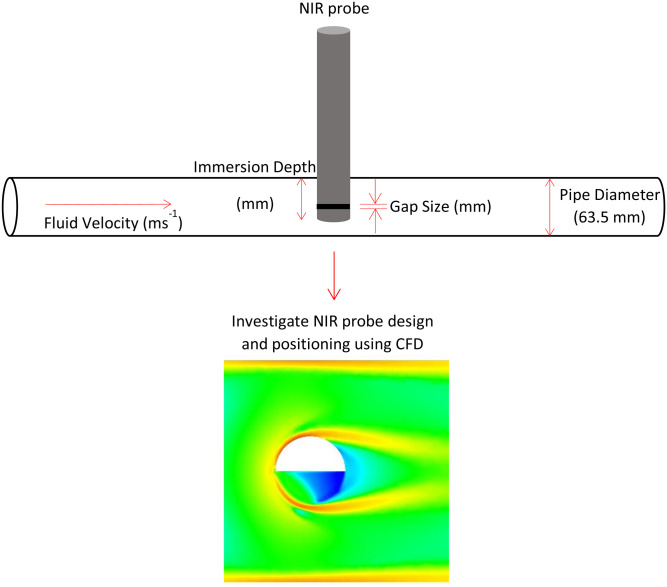

## Introduction

Process analytical technology (PAT) is becoming widespread across manufacturing industries to analyze, measure, and monitor critical quality attributes and process parameters. PAT was first introduced by the U.S. Food and Drug Association (FDA) for the pharmaceutical industry to implement a Quality by Design structure ensuring that processes can produce quality products consistently. Being able to measure critical process parameters and quality characteristics throughout manufacture should lead to a better understanding of the mechanistic principles of the process. The concept is to begin with a “right first time” approach also ensuring processes are compliant with the associated regulations, that is, current good manufacturing practices and good laboratory practice. Prior to the introduction of PAT, the pharmaceutical industries were experiencing product shortages due to batch failures and rejections as well as frequent process irregularities leading to many inconclusive inquiries. With the implementation of PAT, manufacturing productivity and efficiency are being continually improved whilst products are consistently safe and of a high standard.

Process analytical technology has expanded significantly since its introduction in the pharmaceutical industry. Inline and online analytical probes and measuring devices are commercially available, replacing the use of off-line quality control measurements that are usually laborious and time intensive. As outlined in the FDA’s guidelines, the use of PAT instrumentation should not influence the process itself and consequently should have no effect on the product. The design and location of the instrumentation should also ensure representative measurements are made.^1^

The best way to ensure the instrumentation adheres to these guidelines is to optimize its design and setting to understand if varying the placement or fluctuating process variables will affect the measurement and whether there may be an overall ideal design or location. This is useful not only in ensuring accurate and precise measurements but also in aiding calibration transfer, that is, knowing whether there are a set of specifications to follow in terms of probe placement when implementing the same technology in a different factory or plant.

The difficulties that arise with PAT instrumentation will vary based on the application. Accounting for these issues via a trial and error empirical approach to design and optimize the system for the specific application can be time consuming. Alternatively, use of physical models such as computational fluid dynamics (CFD) can be used to simulate the behavior of these systems. CFD plays a role in many industries from engineering noise control around cars^2,3^ to forecasting the weather and arrival of natural disasters.^4^ By solving the Navier–Stokes equations that describe the relationship between velocity, pressure, temperature, and density of a fluid in motion, a visual representation of the moving fluid can be simulated. Unlike the empirical method, this type of mechanistic modeling allows for the prediction, manipulation, and realization of the desired fluid dynamics without the need for experimental work.^5^ Using simulations is extremely useful especially when working with non-Newtonian fluids whose rheology is a function of shear rate that is affected by flowrate changes and varies radially. An in depth discussion of the theory of CFD will not be discussed in this work; therefore, the reader is guided towards works by Tu et al.^6^ and Magoules.^7^

Although introduced in the pharmaceutical industry, PAT is becoming widespread across many manufacturing industries. One important critical quality attribute for many products like creams, shower gels, and paint is viscosity. The viscosity of these products will affect customer satisfaction and functionality. Viscosity is also a key operating parameter which can have significant effects on process efficiency. Unnecessarily viscous products create time and energy inefficiencies that can sometimes result in batch rejections. Therefore, even when the final product viscosity is not important in terms of quality, it is still essential to monitor and control viscosity to ensure optimum process efficiency.

For non-Newtonian fluids in particular, measuring viscosity in-line or on-line is a difficult task to achieve universally. All fluids categorized as non-Newtonian present nonlinear behaviors including shear thinning and shear thickening fluids that exhibit opposite behaviors upon application of a stress, for example toothpaste will become less viscous when subject to an external force, whereas corn starch will become more viscous. This is further complicated when these fluids also present a time dependency, like many paints where they are initially shear thinning, allowing them to be spread. Upon removal of the spreading force, they begin to revert back to their original viscosity over a period of time ensuring the paint does not sag but sits uniformly. Therefore, processing these materials comes with many challenges due to the complex nature of these flows and the difficulty in finding and implementing a suitable and robust technique that can produce representative measurements which have been shown to clearly relate to off-line analysis.^8^ A novel method of determining the viscosity of micellar liquids using NIR spectroscopy has previously been published by our group.^9,10^ This work demonstrates the use of a partial least squares (PLS) calibration model to predict the viscosity of micellar liquids using an inline NIR immersion probe. Other works using NIR to measure viscosity include studies on chocolate,^11^ starch in gravy,^12^ and pharmaceutical cream.^13^

As discussed above, a few studies have shown that the design and/or placement for in-line measuring systems can affect the measurements.^5,14,15^ Therefore, this work looks to use CFD to understand whether the placement of the probe and the effect of varying process parameters effects the viscosity of the process fluid. As the probe being used is a transmission probe with a sample gap of 2 mm, it is important to understand if and how the fluid is sheared as it enters, flows through, and exits the gap. This is important in cases where the process fluid is non-Newtonian as the viscosity is known to vary with shear rate. In order to understand how and if the geometry of the probe might affect the results, immersion depth and angle were also assessed to determine whether there is an ideal configuration.

**Figure 1.**
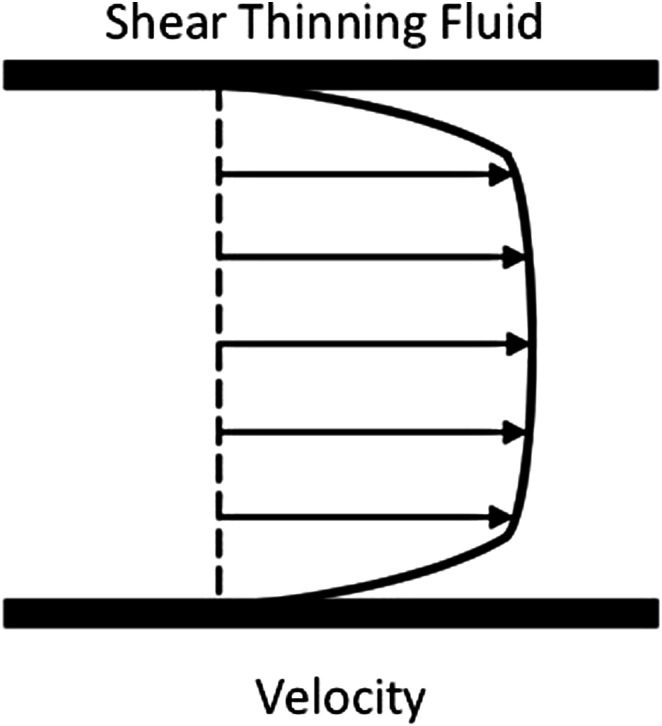
Velocity profile of shear thinning fluids.

For this study, the process fluid is a micellar liquid representative of a cross model fluid or a shear thinning fluid. The velocity profile of shear thinning fluids is shown in Fig. 1. They exhibit profiles reminiscent of flattened parabolic profiles. As the strain exerted on the fluid changes through the cross section of the pipe, the viscosity of the fluid will therefore vary throughout the pipe. The parameters being assessed are immersion depth, angle of immersion, fluid velocity, and gap size.

## Materials and methods

### Computational Fluid Dynamics Model of the Flow

**Figure 2.**
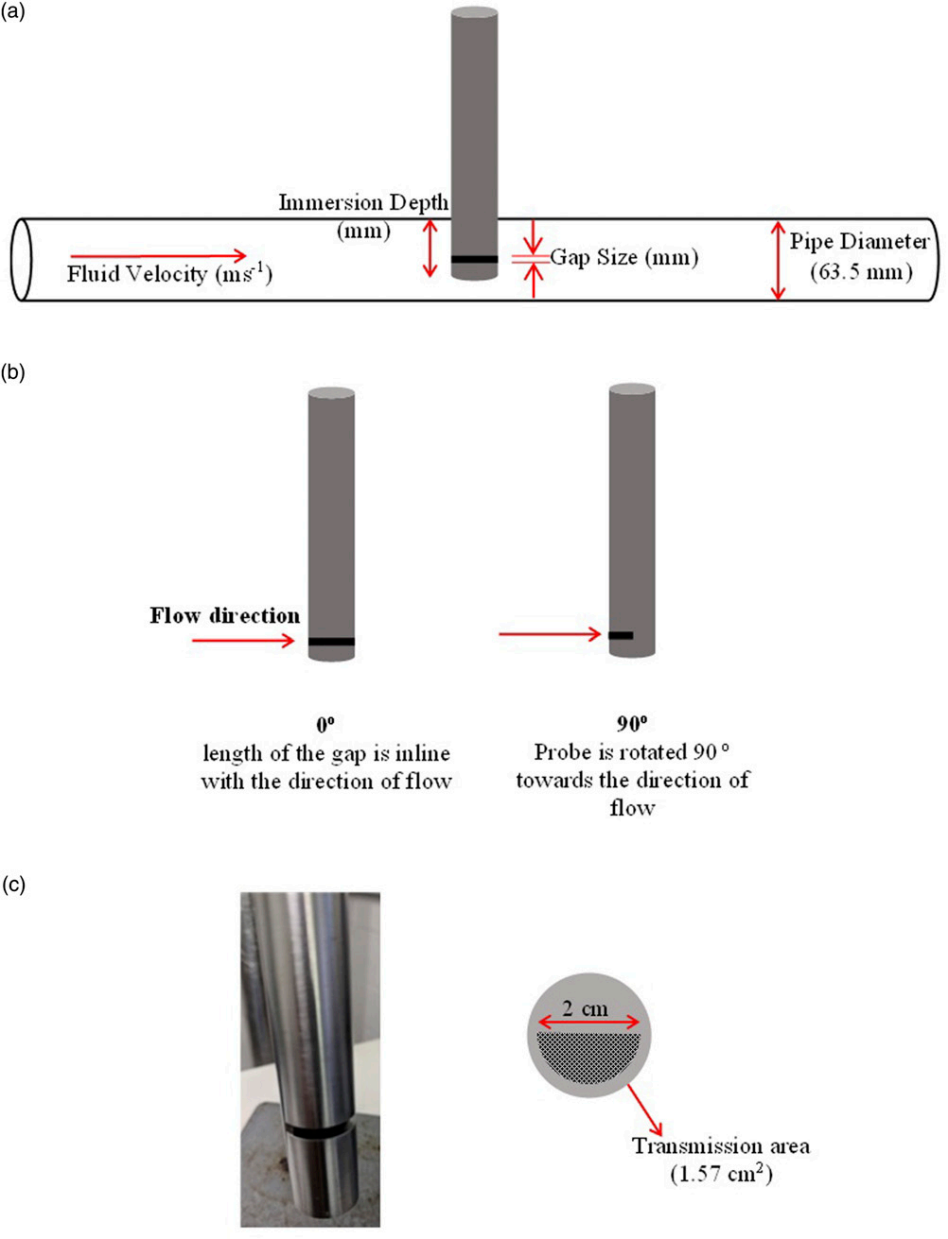
(a) Schematic of NIR probe setup displaying the various parameters being investigated, (b) schematic of immersion angle where the probe is rotated in the radial direction only, and (c) image of NIR probe and schematic of the cross section of the measuring gap.

A computational model of the flow in the pipe around the probe was developed with the commercial finite volume code Fluent v.18.1 (Ansys Inc.). Figure 2 shows a schematic representation of the computational domain and of the measuring interface, showing a transmission based NIR probe (Hellma Excalibur XP 20) immersed in a pipe section. The flow domain was discretized with 2 million tetrahedral elements of variable size to capture all the features of the flow, especially around the probe and in the gap. A mesh independence study was performed beforehand to ensure the results were independent of the level of refinement.

The rheological behavior of the fluid was described with the Cross constitutive equation. Table I shows the Cross model parameters used in this work. The no slip boundary condition was applied at the walls of the pipe and the probe. A uniform inlet velocity was imposed at the inlet boundary condition and a uniform pressure was imposed at the outlet boundary condition. Table I also summarizes the numerical schemes used in this work. The simulations were considered converged when residuals of the order of 10^–^^14^ were achieved.

**Table I.** Settings used in Fluent v.18.1 to perform the simulations.

General settings	
Solver type	Pressure-based
Velocity formulation	Absolute
Time	Steady
Material settings	
Viscosity model	Cross
Zero shear viscosity	8.3 kg (ms)^−1^
Power law index	−0.368
Time constant	0.04899 s
Density	1000 kg·m^−3^ (constant)
Solution method settings	
Pressure–velocity coupling	Coupled (pressure-velocity)
Spatial discretization gradient	Least square cell-based
Spatial discretization pressure	Second order
Spatial discretization momentum	Second‐order upwind

### Parameter Optimization

Figure 2 provides visual guidance showing the parameters being investigated in this study**.** As each parameter was varied, all other variables remained constant (as shown in Fig. 2). The immersion depths were trialed at three points: 32 mm, 57.75 mm, and 63.5 mm. The angle of immersion refers to the angle of the slot in reference to the direction of flow which was trialed at 0°, 15°, 30°, 45°, 60°, 75°, and 90°. Fluid velocity was trialed at 0.5 ms^−1^, 1.0 ms^−1^, and 1.5 ms^−1^ and the measuring gap 1 mm, 2 mm, and 4 mm.

### NIR Experimental Set Up

**Figure 3.**
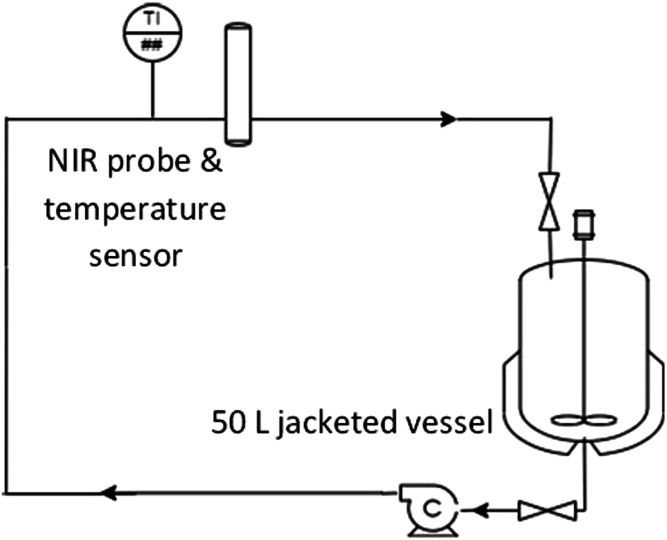
Experimental set up used to validate computational fluid dynamics simulations and 50 L recirculation rig with the in-situ NIR probe and a temperature sensor.

Figure 3 shows the set up of the inline NIR probe in a pilot scale recirculation rig used to validate some of the CFD simulations explored in this study. The spectra were acquired with a Matrix-F Fourier transform near-infrared (FT-NIR; Bruker, Germany) fiber-coupled to a transmission process probe with a pathlength of 2 mm (Excalibur XP 20) where the infrared radiation is transmitted once through the sample. The process fluid was a micellar solution^16^ that was circulated at a constant flowrate of 140 kg·hr^−1^ and temperature of 30 °C ± 2°C. More details related to probe settings, material being measured, and the predictive model used in this work are explained in detail in previous work.^16^

## Results and discussion

### Immersion Angle

**Figure 4.**
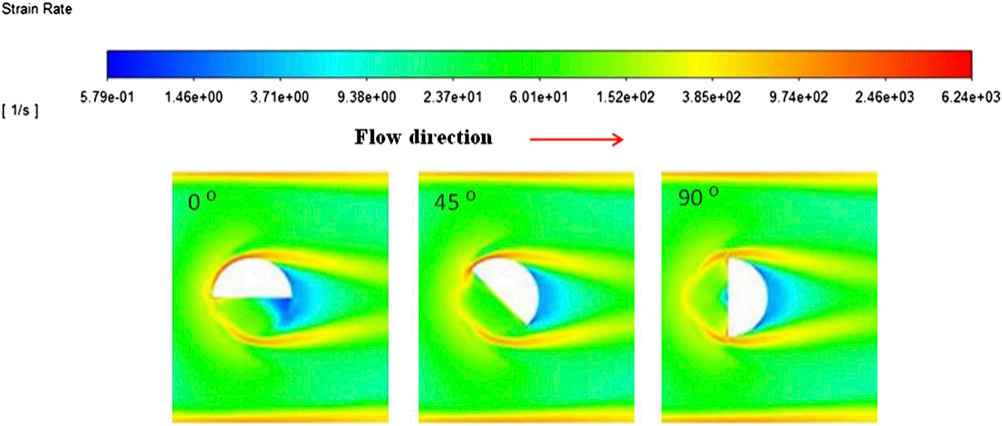
Strain contour plots showing a cross section of the probe across the slot in the pipe with varying immersion angles. At 45 ^o^, the least variation in strain is seen in the measuring gap. The regions of low strain (blue) are likely not being replenished as continually as the fluid in the rest of the measuring gap.

The immersion angle refers to the position of the probe relative to the direction of the flow. Seven different angles were trialed between 0 and 90°, where at 0° the length of the gap is directly in line with the flow. The strain contour plots for 0, 45, and 90° are presented in Fig. 4. All three plots present regions of low strain at the furthest end of the probe (shown in dark blue) in relation to the direction of flow. These regions likely represent stagnant fluid that is not being continually replenished. A horseshoe shaped region of high to very high strain surrounds the probe in all cases as expected due to the obstruction of flow (shown in yellow-red). The high levels of strain are also seen at the walls of the pipe where a no slip boundary condition is assumed. The main difference at each angle displayed is the amount of stagnant fluid in the gap. The largest area of fluid experiencing low strain is present at a position of 0°, at 90° a very small amount appears in the center back of the measuring gap, and no areas of low strain are observed in the measuring gap at 45°.

**Figure 5.**
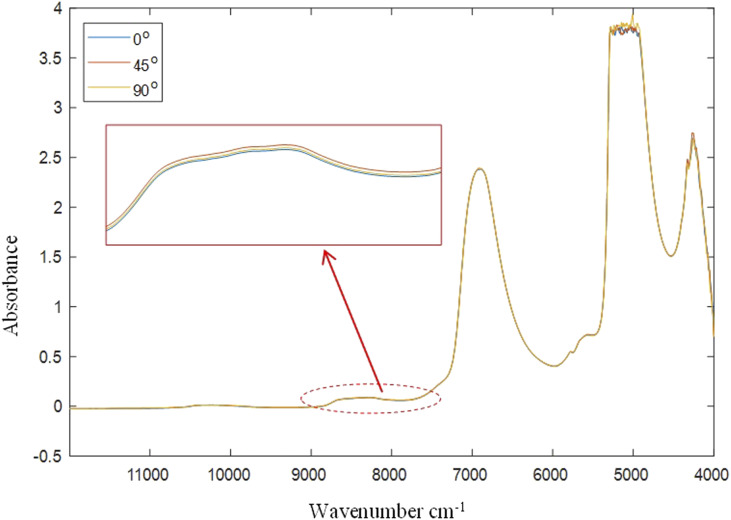
NIR spectra of a micellar solution measured at immersion angles of 0, 45, and 90^o^ showing maximum absorbance intensity at 45°.

From a spectral measurement point of view, stagnant, non-replenished fluids will add inaccuracies to the spectra and consequently to any predictive models as the sample measured would not be representative of the bulk. Figure 5 depicts spectra of a micellar liquid acquired at angles 0, 45, and 90°. At 45°, the absorbance is at its highest which is likely related to changes in the local density variation, reiterating what is seen in the strain contour plots (Fig. 4) where at 45° the sample in the measuring gap is the most consistent with the bulk. As the changes in absorbance are very small, this was repeated three times where each time the same trend as that seen in Fig. 5 was observed.

### Immersion Depth

**Figure 6.**
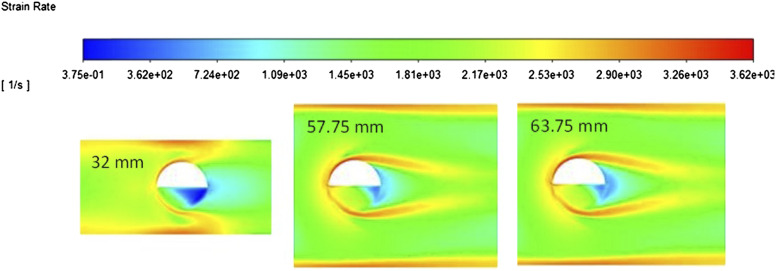
Strain contour plots with varying immersion depths (note as these are viewed from above the contour naturally changes width as the immersion depth changes).

The immersion depth was trialed at three points: the minimum allowed depth as set by the manufacturers (Hellma Analytics, Germany) (32 mm), the maximum possible depth as dictated by the design of the probe and the pipe diameter (63.75 mm), and directly in the center of the flow (57.75 mm). The strain contour plots (Fig. 6) around the interface of the probe are similar at depths of 57.75 mm and 63.75 mm. Due to the nature of the shear thinning flow profile having a blunt top, it is likely that the fluid being analyzed at each of these immersion depths is flowing at the same or very similar velocities. This is not the case at a depth of 32 mm where the probes measuring gap is closer to the pipe wall. Here, the fluid experiences higher levels of strain as a no slip boundary condition is assumed. It is important to note that at the higher immersion depths, the majority of the fluid in the measuring gap experiences a similar strain to that shown in the bulk. However, at 32 mm, there is much more variation in the strain rate across the measuring gap with a large proportion of fluid experiencing very low levels of strain likely to be stagnant as discussed in the section, Immersion Angle, below.

### Measuring Gap

**Figure 7.**
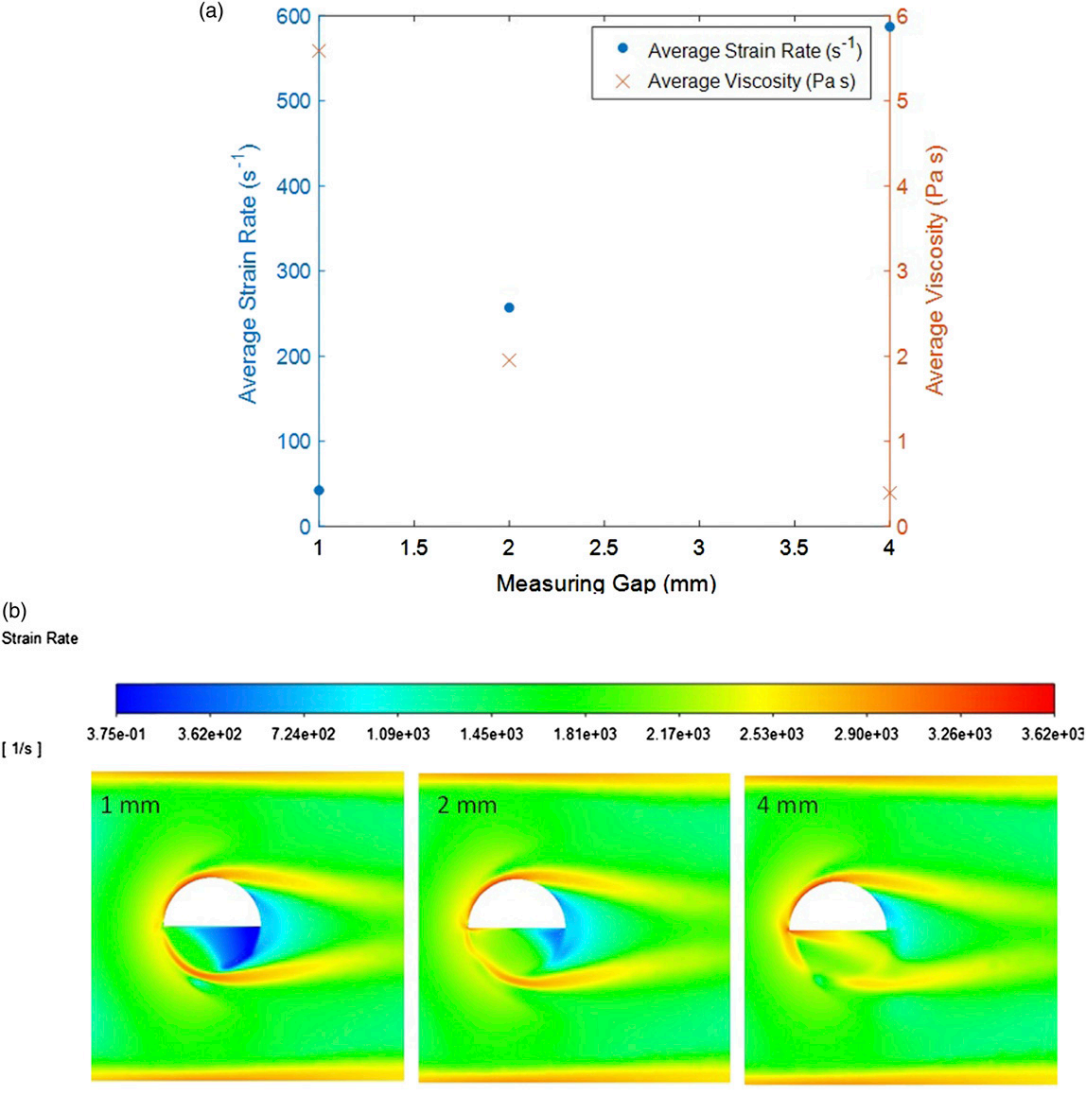
(a) Average strain rate and viscosity in the measuring gap with varying measuring gap size determined by computational fluid dynamics, (b) strain contour plots with varying measuring gaps (left:1 mm; center: 2 mm; right: 4 mm).

The probe being modeled has a measuring gap of 2 mm (Fig. 2). This gap was varied (1 mm and 4 mm) using simulations to see whether there is an ideal gap size for this application. Figure 7a shows the average viscosity and strain rates in the measuring gaps with changing size. It was predicted that the smaller the gap size, the larger the average strain and the lower the average viscosity. However, the opposite is found to occur in this case where the average strain rate increases with increasing gap size. To understand this, Fig. 7b presents the strain contours for each measuring gap showing that more stagnant fluid is present as the gap gets smaller. The horseshoe shape of high strain around the probe in all cases clearly shows the fluid would rather flow around the probe and in the case of the 1 mm gap is unable to flow fully through the measuring gap. Whereas at 4 mm the fluid flows more easily through the gap where no stagnant fluid is present. Instead, there is a region of high strain adding to the non-uniformity of the sample in the gap which also explains the large decrease in viscosity shown in Fig. 7a. At 2 mm there is a much smaller area of stagnant fluid in the gap compared with the 1 mm and overall, it shows that the majority of the fluid in the gap is under the same conditions as the bulk but still presents some inhomogeneity.

As the probe is transmission based, the size of the gap will influence the absorption intensity. The larger the gap, the more sample present and, provided the sample is homogenous, the larger the absorbance. It is unknown whether these effects will influence the PLS model, and this has therefore been investigated using the data set and model developed in. To account for the different gap sizes, the absorbances of spectra collected using the original probe (2 mm) were scaled to the corresponding gap size. Though the absorbances visibly varied with gap size, the model predictions remained the same across all three gap sizes. This suggests that the regions of the spectra being used in this model show proportionality between absorbance and pathlength. Models using parts of the spectra that show nonlinear changes are likely to present differences when varying the pathlength.

### Fluid Velocity

**Figure 8.**
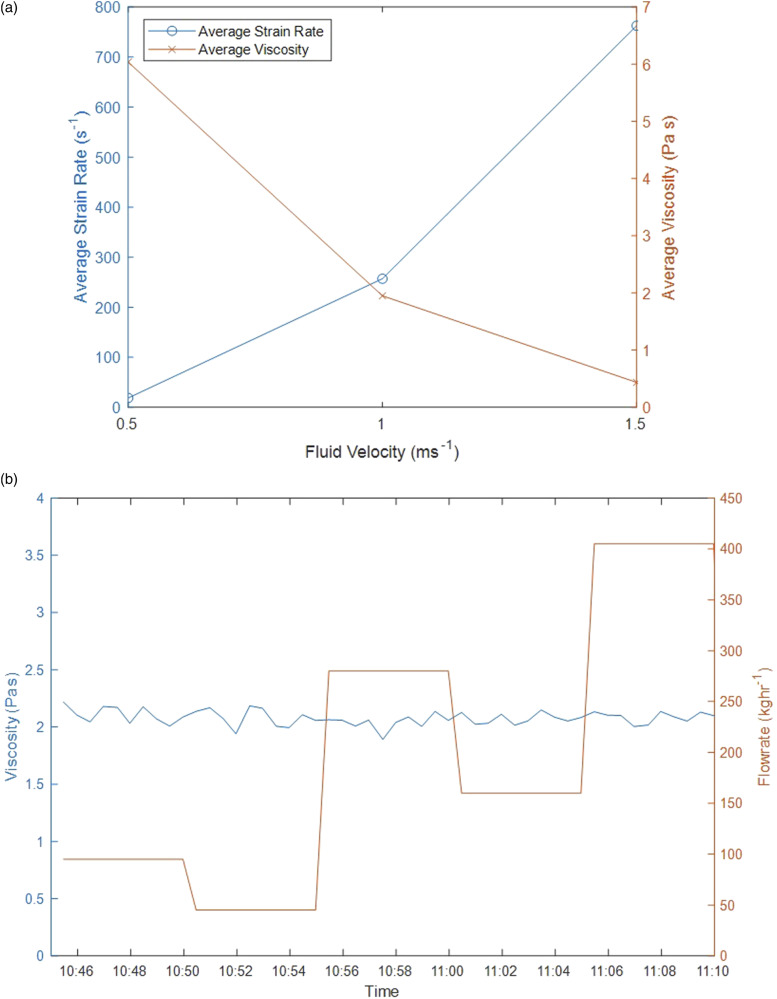
(a) Average strain rate and viscosity in the measuring gap with varying fluid velocity. An increase in average strain rate and decrease in average viscosity is typical of what would be expected for a shear thinning fluid, (b) effect of flowrate on viscosity predictions for a micellar liquid where flowrate was varied systematically and randomly. Clearly, there is no correlation between the predicted viscosities (blue) and flowrate (red), suggesting flowrate does not affect the model.

During production of micellar liquids, the flowrate is fairly consistent though this is not the case in all industries producing non-Newtonian materials. With increased flowrate, the strain exerted on a non-Newtonian fluid increases resulting in changes in fluid viscosity. Therefore, varying fluid velocity was investigated to determine whether these effects translate through to the fluid in the measuring gap of the probe. Fluid velocities were trialed at 0.5, 1.0, and 1.5 ms^−1^. Figure 8a shows the fluid velocity against average strain rate and average viscosity in the measuring gap where with increased velocity the average strain rate increases, and average viscosity decreases within the measuring gap of the probe. This is consistent with what would be expected for a shear thinning material.

The model used in this work has been shown to be unaffected by changes in flowrate. Figure 8b shows how changing the flowrate effects the predictions from the model where data was collected in-line using a pilot scale setup. Clearly, there is no relationship between the two suggesting the model focuses only on information related to absorbance. This may not be the case for all applications; therefore, dependent on whether the model is produced using the NIR spectra based on absorbance, scattering, or both, the flowrate will need to be considered, perhaps by including it during model development or correcting for it at a later stage.

## Conclusion

Many process analytical techniques use a probe interface to the process and there is little reported work in the literature on their optimal design and implementation in process applications ensuring they work as expected providing accurate and precise data. This study shows the importance of doing so for an in-line NIR transmission probe for the measure of viscosity of a shear thinning fluid. The parameters investigated can be split into interfacing (immersion depth, angle, and light transmission gap) and process variables (fluid velocity). These present only a few of the variables that would be of interest when implementing such technologies for the first time as well as being useful in terms of calibration transfer, knowing what will affect the measurements and making the transfer process faster and more transparent. CFD modeling would also be beneficial when designing in-line analytical instruments to ensure that they are fit for purpose.

For the application of micellar liquid viscosity, it was found that immersion depth of the probe should be far enough away from the pipe walls and within the flattened part of the shear thinning profile to try and ensure that the fluid that enters the gap is experiencing similar shear. The immersion angle was found to be critical in relation to the amount of low strain/assumed stagnant fluid present in the measuring gap The ideal angle was found to be 45° as no stagnant fluid was shown to be present. Varying the transmission gap showed no differences in the predictive models developed for this work. Although, it is likely to be more advantageous when using parts of the spectra that are nonlinear and also would be more practical when dealing with more viscous materials. Finally, variation in fluid velocity produced the expected results for a shear thinning material and so should either be controlled well or accounted for during model development. Temperature was not investigated in this study but is known to effect viscosity and therefore would be a useful parameter to study in future work. The location and positioning of the probe in the process (i.e., in a pipe bend) would also be of interest as this study focuses on the probe positioned in the center of a horizontal pipe.
